# Responses of Soil Rare and Abundant Sub-Communities and Physicochemical Properties after Application of Different Chinese Herb Residue Soil Amendments

**DOI:** 10.4014/jmb.2202.02029

**Published:** 2022-03-16

**Authors:** Fan Chang, Fengan Jia, Min Guan, Qingan Jia, Yan Sun, Zhi Li

**Affiliations:** 1 College of Life Science, Shaanxi Normal University, Xi’an 710062, P.R. China; 2 Shaanxi Institute of Microbiology, Xi’an 710043, P.R. China; 3 Shaanxi Agricultural Machinery Research Institute, Xianyang 712000, P.R. China; 4 Institute of Medical Research, Northwestern Polytechnical University, Xi'an 710072, P.R. China

**Keywords:** Rare sub-community, abundant sub-community, soil amendments, bacterial communities, soil physicochemical properties

## Abstract

Microbial diversity in the soil is responsive to changes in soil composition. However, the impact of soil amendments on the diversity and structure of rare and abundant sub-communities in agricultural systems is poorly understood. We investigated the effects of different Chinese herb residue (CHR) soil amendments and cropping systems on bacterial rare and abundant sub-communities. Our results showed that the bacterial diversity and structure of these sub-communities in soil had a specific distribution under the application of different soil amendments. The CHR soil amendments with high nitrogen and organic matter additives significantly increased the relative abundance and stability of rare taxa, which increased the structural and functional redundancy of soil bacterial communities. Rare and abundant sub-communities also showed different preferences in terms of bacterial community composition, as the former was enriched with *Bacteroidetes* while the latter had more *Alphaproteobacteria* and *Betaproteobacteria*. All applications of soil amendments significantly improved soil quality of newly created farmlands in whole maize cropping system. Rare sub-communitiy genera *Niastella* and *Ohtaekwangia* were enriched during the maize cropping process, and *Nitrososphaera* was enriched under the application of simple amendment group soil. Thus, Chinese medicine residue soil amendments with appropriate additives could affect soil rare and abundant sub-communities and enhance physicochemical properties. These findings suggest that applying soil composite amendments based on CHR in the field could improve soil microbial diversity, microbial redundancy, and soil fertility for sustainable agriculture on the Loess Plateau.

## Introduction

The application of plant residues is of great importance due to its positive effect on both crop and soil health [1]. The incorporation of plant residues generally results in increased soil organic matter, enzyme activities [2] and essential nutrient retention capacity [3]. Similar to crop straw and green manure, Chinese herb residue (CHR) is a solid plant residue left over from processing extraction of ingredients [4]. It was reported that the incorporation of CHR soil amendment improved soil aggregate stability and enhanced agro-ecosystem sustainability [5]. As an environmentally friendly approach, the addition, after fully composting, of spent mushroom substrate or goat manure, which contain large amounts of nitrogen source and organic matter, has been recommended to promote crop growth and increase crop yield in agricultural ecosystems [6, 7].

The application of soil amendments has shown different effects on soil microbial diversity and structure [8]. Soil microbial communities play an important role in nutrient cycling of different agricultural systems [9]. These communities commonly exhibit unbalanced distributions, whereby they contain a large number of low-abundance taxa and a small number of high-abundance taxa [10]. The highly abundant microbes that account for the majority of microbial biomass are thought to be most important in carbon cycling [11]. However, recent studies have increasingly emphasized the importance of rare taxa and investigated the responses of rare sub-communities to different cropping systems [12, 13]. Currently, little is known about the responses of soil bacterial abundant and rare taxa under the application of soil amendments.

Cropping systems generally result in improved soil physical and chemical properties and shaped microbial soil communities [14]. Studies have emphasized the importance of the soil-borne microbiome in maintaining agricultural sustainability [15, 16]. Studies on soil amendments generally select simple components [17, 18]. However, there are few studies investigating the effects of exogenous composite soil amendments and cropping systems on soil rare and abundant sub-communities.

The loess soil in the Loess Plateau of China has poor water-holding capacity, and the organic matter content of newly created farmland is low [19]. As one of the major grain production regions of dry-land farming in China, choosing the optimum soil amendment strategy is a crucial step for maintaining soil quality in agricultural areas of the Loess Plateau. In the present study, we aim to (i) explore the patterns of rare and abundant bacterial taxa under different CHR soil amendments in dry-land cropping systems and (ii) uncover the soil properties regulating the diversity and structure of rare and abundant bacterial taxa. For this purpose, the soil micro-ecological effects were investigated in newly created farmland after the application of different soil amendments on the Loess Plateau.

## Materials and Methods

### Different Soil Amendments and Sample Collection

The simulated open composting of Chinese herb residue (CHR) was carried out at the Shaanxi Institute of Microbiology, Xi’an, Shaanxi, China [20]. The CHRs were collected after 60 days. Briefly, two main soil amendments with CHRs were designed: Simple soil amendments (Simple) and composite soil amendments (Composite). CHRs (CHR) and CHRs with urea (CHR_U) amendments were used as simple soil amendments. CHRs with spent mushroom substrate (CHR_SMS) and CHRs with goat manure (CHR_GM) were used as soil amendments. The carbon/nitrogen ratio was regulated similarly for all groups other than CHRs group.

The soil amelioration field trial was established in April 2020 and was located at the village of Nangou in Yan'an, Shaanxi Province, China (36°35'26"N 109°18'47"E). The soil texture of the newly created farmland was mainly fine loess. This area is characterized by a plateau continental monsoon climate. The average annual temperature ranges between 7.7-10.6°C. The average precipitation is 490.5-663.3 mm, with nearly 70% occurring between the months of July and September [21]. The experimental plots were arranged in a randomized complete block design with three replicates. Two main soil amendments with four different treatments (~6,000 kg/ha) were applied as base fertilizers for the 0–20 cm soil layers prior to planting. Maize (*Zea mays*) seeds were sown at a density of 20 kg/ha. No additional fertilizers were applied during plant growth.

All soil samples were collected in April and August 2020, after one month of maize sown in spring and before maize harvest in autumn. Three replicates, collected for each separate block, were collected for three biological replicates. Finally, 30 samples of surface soil (0- to 20-cm depth) were collected from each block, sealed, transported at 4°C and stored at −80°C until DNA was extracted.

### DNA Extraction, Amplification, and Sequence Processing

DNA from 30 samples collected from the different blocks was extracted using the E.Z.N.A. Stool DNA Kit (D4015, Omega, Inc., USA) according to manufacturer’s instructions. This reagent was designed to uncover DNA from trace amounts of sample and has been shown effective for isolating DNA from most bacteria. Nuclear-free water was used as a blank control. Total DNA was eluted in 50 μl of elution buffer and stored at -80°C until subjected to PCR using LC-Bio Technology Co., Ltd. (China).

The V3 and V4 hypervariable regions were selected for generating amplicons and subsequent taxonomy analysis for prokaryotic 16S rDNA. The V3 and V4 regions were amplified using the following primers: 341F (forward primer 5'-CCTACGGGNGGCWGCAG-3') and 805R (reverse primer 5'-GACTACHVGGGTATCTAATCC-3') [22]. PCR amplification was performed in a 25 μl reaction mixture containing 25 ng of template DNA, 12.5 μl of PCR Premix, 2.5 μl of each primer and PCR-grade water. The PCR conditions to amplify the prokaryotic 16S fragments consisted of an initial denaturation at 98°C for 30 sec; 32 cycles of denaturation at 98°C for 10 sec, annealing at 54°C for 30 sec, extension at 72°C for 45 sec and final extension at 72°C for 10 min. PCR products were run on a 2% agarose gel. Throughout the DNA extraction process, ultrapure water, instead of sample solution, was used to exclude the possibility of false-positive PCR results. PCR products were purified using AMPure XT beads (Beckman Coulter Genomics, USA) and quantified using Qubit (Invitrogen, USA). Amplicon pools were prepared for sequencing, and the size and quantity of the amplicon library were assessed on the Agilent 2100 Bioanalyzer (Agilent, USA) and using the Library Quantification Kit for Illumina (Kapa Biosciences, USA). Libraries were sequenced using a NovaSeq PE250 platform. These sequences are available on the National Center for Biotechnology Information (NCBI) (https://www.ncbi.nlm.nih.gov/bioproject/PRJNA691996), under the study accession no. PRJNA691996.

### Detection of Soil Physicochemical Properties

Samples were air-dried in a drying room. Organic matter (OM), total N (TN), total phosphorus (TP), Olsen-P (AP), total potassium (TK), soil moisture content (H_2_O), soil electrical conductivity (EC) and pH were measured. Organic matter was measured using sulfuric acid-potassium dichromate wet oxidation [23]. TN was determined using the Kjeldahl method [24]. TP in the soil was measured using the Mo-Sb anti spectrophotometric method [25]. AP in the soil was measured using the Olsen method [26]. TK in the soil was determined using a flame photometer after ammonium acetate extraction [27]. Soil moisture content was determined gravimetrically (drying at 105°C for 48h). Soil electrical conductivity was determined by the electrical conductivity method (soil: water = 1:5). Soil pH was measured by a pH electrode (soil: water = 1:2.5) [28].

### Data Analysis and Statistical Analysis

The Usearch10 and Vsearch 2.8.1 (Rognes *et al*. 2016) data analysis pipelines were used to analyze 16S rRNA data. Forward and reverse reads were joined, assigned to samples based on barcodes and truncated by removing the barcode and primer sequences. Quality filtering was performed on joined sequences. Sequences that did not fulfill the following criteria were discarded: no ambiguous bases and expected errors per base rate > 0.01. dereplicated and singletons sequences (size < 8). Then, sequences were clustered into amplicon sequence variants (ASVs) using the Unoise3 sequence variants algorithm [29, 30]. Chimeric sequences were simultaneously removed. Effective sequences were used in the final analysis. The 16S sequences were grouped using the clustering program VSEARCH 2.8.1 (Rognes *et al*. 2016) against the Ribosomal Database Program (RDP, http://rdp.cme.msu.edu/), and preclustered at a sequence identity of 97%. The classifier [31] was used to assign taxonomic categories to all ASVs using a confidence threshold of 0.8.

The ‘rare’ ASVs were defined as those having relative abundances below 0.01% of total sequences and ‘abundant’ ASVs as other sequences [12, 32]. All statistical analyses were performed using MicrobiomeAnalyst [33, 34](https://www.microbiomeanalyst.ca/) and R software (v4.1.2; http://www.r-project.org/). Alpha-diversity was evaluated using richness, Shannon, and Pieloús evenness diversity indices based on the relative abundance table via the alpha_div of Usearch10. The effects of different treatments and seasons on bacterial alpha-diversity were calculated using one-way analysis of variance (ANOVA) and Tukey's multiple comparisons test. Principal coordinate analysis (PCoA) plots were generated from a Bray-Curtis dissimilarity matrix of ASVs and Permutational multivariate analysis of variance (PERMANOVA) analysis was performed from the R package ‘vegan’ [35]. *Proteobacteria* was divided into four subphyla, and a stacked bar chart at phylum level was drawn to check relative abundance. The ternary diagram with ‘ggtern’ package of R [36] was used to show the enriched and differentiated genus of treatments. The correlation between the genus and soil properties variables was assessed by redundancy analysis (RDA) using the ‘vegan’ package in R. Analysis of variance inflation factors (VIF) was performed on final covariates to confirm acceptably low multicollinearity (VIF<10). PERMANOVA based on Bray–Curtis distances was used to test for significant differences between groups. The *envfit* function tests was used to calculate significance of association between environmental factors and genus community.

## Results

### Bacterial Sequencing Analysis and General Patterns of Rare and Abundant Taxa

A total of 1,218,239 raw reads were obtained from 30 samples. The samples yielded 307,477 high-quality sequences (average of 10,249 ± 124.11 per sample) after filtering and chimera removal. The high-quality reads were clustered into 2611 microbial ASVs. The average coverage was 96.08 ± 1.56% for the total bacterial community.

**Fig. 1:**
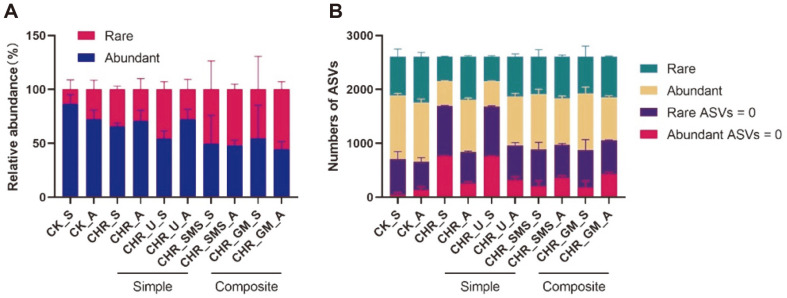
The relative abundance and number of rare and abundant ASVs in different soil treatments. **A**. The relative abundance of rare and abundant ASVs. The red bars represent the rare ASVs, and the blue bars represent the abundant ASVs. **B**. The number of rare and abundant ASVs. The green bars represent the the number of rare ASVs, the yellow bars represent the number of abundant ASVs, and the dark bule and pink bars represent no ASVs observed. Simple: CHR and CHR_U treatments; Composite: CHR_SMS and CHR_GM treatments. Spring and Autumn are represented by the letters S and A.

There were about the same number of both rare and abundant ASVs, but the relative abundance of rare taxa under soil treatments showed different patterns across maize planting (Figs. 1A and 1B). The number of abundant ASVs was between 457 to 1220, and that of abundant ASVs was between 449 to 936. No significant difference in four treatments was observed between rare and abundant taxa across maize planting. With different treatments, the relative abundance of soil rare taxa was increased. Trends in rare and abundant sub-communities were opposite in spring and antumn. Rare ASVs in simple soil amendments were higher (48.75% ~ 79.16%) in the spring, with opposite trends for those in composite soil amendments (54.72% ~ 61.91%).

### Soil Bacterial Diversity of Rare and Abundant Sub-Communities

**Fig. 2:**
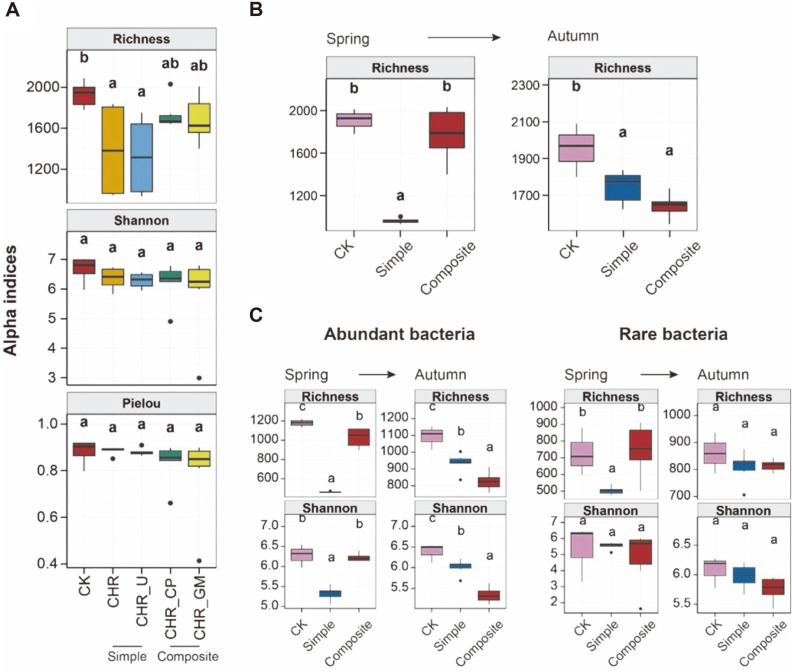
Richness, diversity and evenness in different soil treatments. **A**. Richness, Shannon, and Pielou’s evenness indices with different soil treatments. **B**. Richness index in different seasons with simple and composite soil amendments. **C**. Richness and Shannon indices of the abundant and rare sub-communities in different seasons with two amendments.

The simple soil amendments significantly decreased the bacterial richness index, but did not affect bacterial evenness (Shannon index) and diversity (Pieloús evenness index) (Fig. 2A). In spring, there were no significant differences in bacterial richness between CK and composite soil amendments groups. However, the simple soil amendments bacterial richness significantly reduced. In autumn, the simple and composite soil amendments showed no significant differences in bacterial richness, and differed significantly from CK group (Fig. 2B), indicating that the difference is mainly caused by the treatments of the simple soil amendments in spring.

Significant richness and evenness differences were observed for the abundant sub-communities in autumn for the simple and composite amendments. There were no significant differences between amendments for the rare sub-communities, same as for the soil bacterial communities (Figs. 2B and 2C). This indicated that the rare sub-communities determined the bacterial diversity, while the abundant sub-community diversity was more active.

### Soil Bacterial Community Structures and Shift under the Soil Amendments

**Fig. 3:**
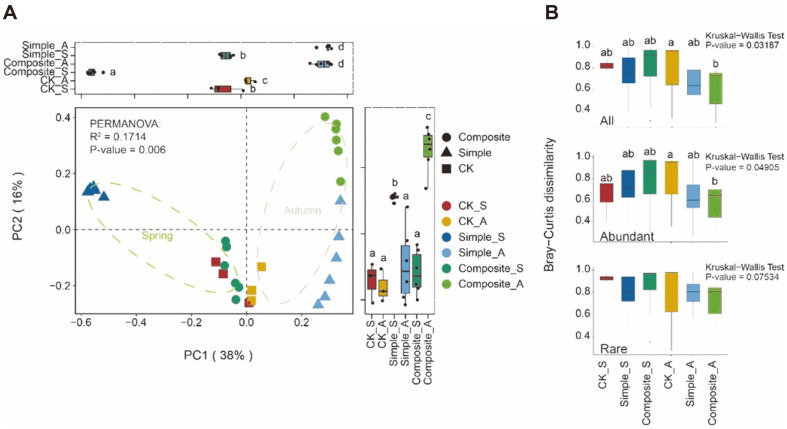
A. PCoA plot of Bray–Curtis distances between samples (54% of variance, *p* = 0.006). B. Bray-Curtis dissimilarity index boxplot between different amendments in spring and autumn.

The Bray–Curtis distances between samples using PCoA to determine how dissimilar the soil bacterial community structures were in different seasons with soil amendment treatments. Unconstrained ordinations revealed a clear separation of bacteria between spring and autumn. The bacterial communities of different seasons formed two distinct clusters, which separated along the PC1 (Fig. 3A), indicating that the largest source of variation in the soil bacterial community was the stage of cropping process. As expected, the distance of simple soil amendment samples differed from those of composite soil amendments, but the soil bacterial community structures between the two amendments in autumn were clustered along the PC1 while being separated along the PC2 (Fig. 3A). This indicated that bacterial community structures between the two amendments were more similar in autumn.

Dissimilarity in bacterial community structure was calculated using the Bray–Curtis dissimilarity index (Fig. 3B). The bacterial communities of CK group in autumn showed the highest beta-diversity, indicating the highest dispersion. The bacterial communities of simple and composite amendments in autumn showed lower beta-diversity and had no significant difference, indicating their lower dispersion. Furthermore, abundant and rare sub-communities with composite soil amendments had the lowest beta-diversity in autumn.

### Bacterial Composition Changes under the Simple and Composite Amendments

**Fig. 4:**
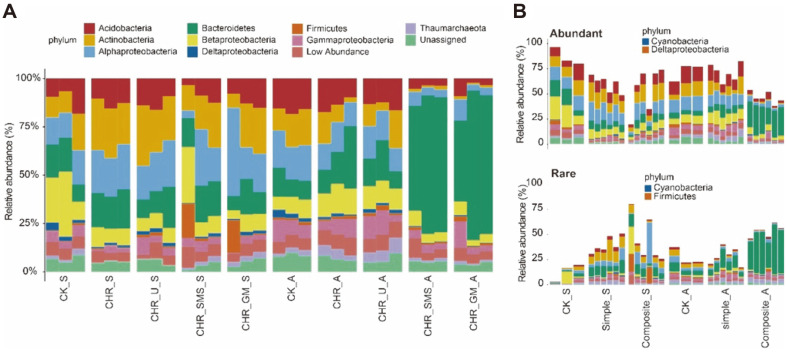
Relative abundances of different bacterial taxa in all samples. **A**. Relative abundance of top 9 bacterial phyla per sample. Low abundance represents the other phyla. **B**. Relative abundance in the community of abundant and rare subcommunities at phylum level. Bacterial phyla were represented by different colors.

Relative abundances of bacterial community composition with different treatments at phylum level were shown in Fig. 4. The top 9 bacterial phyla were shown from all soil samples analyzed in this study. *Acidobacteria*, *Actinobacteria*, *Alphaproteobacteria*, *Bacteroidetes*, and *Betaproteobacteria* were revealed as the top five most prevalent phyla in the microbiota present in all soil samples. Notably, the application of composite amendments changed the bacterial community composition. *Bacteroidetes* was enriched in CHR_SMS and CHR_GM groups in autumn (Fig. 4A).

Abundant and rare sub-communities showed a preference in terms of the bacterial community composition. The relative abundance of *Alphaproteobacteria* and *Betaproteobacteria* in the abundant sub-community was higher. The relative abundance of *Bacteroidetes* was higher in the rare sub-community. *Deltaproteobacteria* was unique to the abundant sub-community, while *Firmicutes* was unique to the rare sub-community. In Composite_A group, both rare and abundant sub-communities were enriched with *Bacteroidetes* (Fig. 4B).

### Bacterial Community Genera Distribution under the Application of Different Soil Amendments

**Fig. 5:**
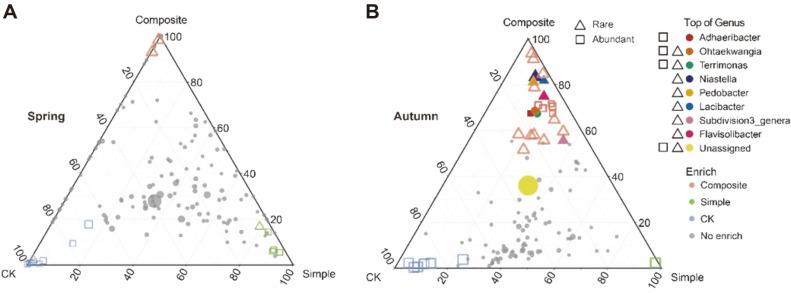
Ternary plot of different genera for CK, simple soil amendment and composite soil amendment groups. **A**. Different genera in spring. **B**. Different genera in autumn. The size of each shape represents the relative abundance. The triangle depicted rare sub-community genus, the square depicted abundant sub-community genus, and color of triangle or square depicted the top 10 genera in relative abundance. Blue, red, and green color represented the enriched genera in simple soil amendment and composite soil amendment groups, respectively.

The ternary plot compared the bacterial genera in two soil amendments and CK groups (Fig. 5). In spring, there was no top 10 genera in relative abundance observed in three groups. Rare and abundant sub-community genera were observed in all three groups (Fig. 5A), indicating that the bacterial communities were a generalist distribution with different soil amendments and had not developed their own dominant species in spring.

When comparing the different bacteria of three groups in autumn, the ternary plot results showed that most of the top genera had a composite soil amendment‐specific distribution. This distribution consisted mainly of rare sub-community genera. The rare sub-community genera *Niastella*, *Pedobacter*, *Lacibacter*, Subdivision3_genera, and *Flavisolibacter* were enriched in the Composite group. Moreover, *Ohtaekwangia* and *Terrimonas* enriched in the Composite group were both in the top 10 genera in relative abundance of the rare and abundant sub-community genera. There were many unassigned genera enriched in the Composite group. Furthermore, many rare sub-community genera with low relative abundance (red triangle) were also enriched in the Composite group. *Adhaeribacter* was the only abundant sub-community genus enriched in the Simple group (Fig. 5B). This result indicated that rare sub-community genera may be unique under the application of composite soil amendments and verified that the rare species distribution pattern was more affected by the composite soil amendments.

### Effect of Soil Physicochemical Properties on Bacterial Community Distribution

The physicochemical characteristics of the different treatment soils were investigated. Overall, all treatments improved soil physical and chemical properties (Table 1). Compared to CK group, the composite soil amendments (CHR_SMS and CHR_MR groups) significantly increased the soil OM, nitrogen, phosphorus, AK, and EC in spring and autumn, while pH was significantly decreased. The simple soil amendments (CHR and CHR_U groups) significantly increased the soil OM, nitrogen and phosphorus in autumn. This result showed that the effects of the composite soil amendments on soil physical and chemical properties may be greater and more persistent than those of the simple soil amendments.

The relationships between bacterial genera and soil physicochemical properties in different application of soil amendments were analyzed by redundancy analysis (RDA). Genera responsive to soil physicochemical properties were fitted onto the ordination (function *envfit*).

**Fig. 6:**
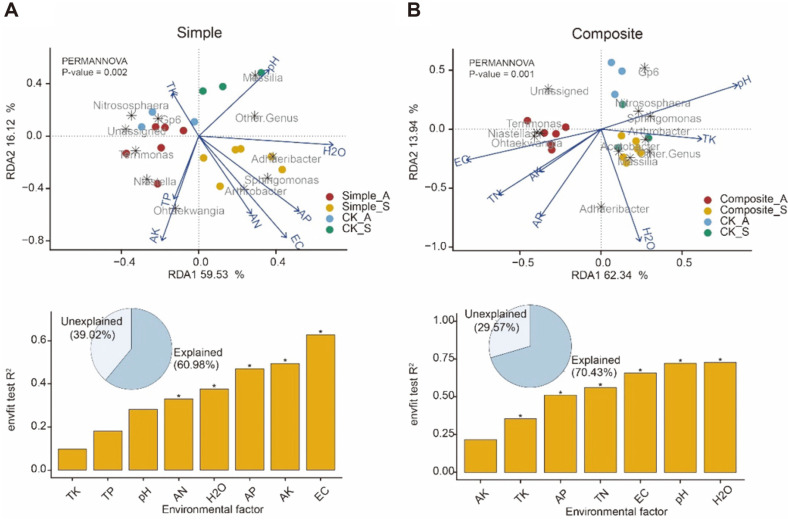
Redundancy analysis between bacterial community structures of genus and soil physicochemical properties. **A**. RDA of the simple soil amendment group and significant effect soil properties. **B**. RDA of the composite soil amendment group and significant effect soil properties.

For the relationship between the soil physicochemical properties and genera distributions in spring and autumn, the cumulative percentage variance explained by the first two canonical RDA axes was 76.40% and 76.28%, respectively. The RDA revealed that EC, AK, AP, soil moisture content, and AN in simple soil amendments group had a greater effect on bacterial community structures of genus. *Adhaeribacter*, *Sphingomonas*, *Arthrobacter* and *Ohtaekwangia* had a positive correlation with the soil properties. *Nitrososphaera*, *Terrimonas* and *Niastella* had a negative correlation with the significant effect soil properties. The bacterial community genera of Simple_S group had a positive correlation with all significant effect soil properties (Fig. 6A). In the application of composite soil treatment group, soil moisture content, pH, EC, TN, AP, and TK had a greater effect on bacterial community structures of genus. *Terrimonas*, *Niastella*, and *Ohtaekwangia* had a positive correlation with EC, TN and AP, while all factors were positively correlated with Composite_S samples. *Adhaeribacter*, *Nitrososphaera*, *Sphingomonas*, *Arthrobacter*, *Acetobacter*, *Massilia*, and Gp6 had a positive correlation with soil moisture content, TK, and pH, and all factors were positively correlated with Composite_A samples (Fig. 6B). This result indicated that bacterial genera under the composite soil amendments were more specific in different seasons.

## Discussion

Composite soil amendments have the potential to restore soil fertility through nutrient cycling, water retention, microbial community, disease suppression and other processes [37]. High-throughput sequencing technologies have provided increasing resolutions to reveal the biospheres of rare and abundant sub-communities [10]. In this study, we investigated the effects of different soil amendments on rare and abundant bacterial sub-communities and soil physicochemical properties in dry-land farming systems. Here, we showed that rare and abundant bacterial diversity exhibited distinct patterns that were driven by different applications of soil amendments. *Ohtaekwangia* and *Niastella* predominately mediated the rare microbial sub-communities in application of composite soil amendments in dry-farming systems. Soil moisture content, EC, and AP had a greater effect on bacterial community structures of genus.

### Effects of Soil Amendments on Bacterial Rare and Abundant Sub-Communities

In the present study, the relative abundance of rare taxa was higher in the soil amendment groups, despite having similar numbers of species (Fig. 1). This trend suggested that rare taxa were mediated by the extraneous substances in agricultural ecosystems. Studies suggested that the abundance of diversity redundancy in rare taxa provided a functionally redundant ‘seed bank’ for soil environmental change, and could become dominant under the proper conditions [38, 39]. Previous reports have found that the composite soil amendments raised the chemical, physical and biological soil properties associated with the addition of organic matter [40]. In our study, the composite soil amendments consisting of Chinese herb residues with additives significantly increased the relative abundance of rare taxa (Table 2). One study found that applying Chinese herb residues in the field could improve soil nutrients and properties for sustainable agriculture on the Loess Plateau [5]. We also found that the application of simple soil amendments (only Chinese herb residues and CHR with urea) had no significant effect on soil relative abundance of bacterial rare and abundant taxa (Fig. 1 and Table 2). The additives of spent mushroom substrate and goat manure could provide plenty of nitrogen, organic matter, and exogenous microbial communities [7, 41], which may be the main reason for the response of soil microbial communities.

In spring, the bacterial richness was significantly decreased under the simple soil treatments. This suggested that a large amount of introduced organic matters could dramatically reduce bacterial diversity in the soil in the short term consistent with previous research [42]. The microbiota in the additives of composite amendments may stabilize the soil bacterial diversity. In autumn, the composite soil amendments reduced the bacterial richness, which was similar to a previous study [43]. Rare taxa could increase soil resiliency to environmental disturbances [44]. In our study the rare sub-communities were more stable and determined the bacterial diversity, while the abundant sub-community diversity was more active in the late maize cropping stage.

### Effects of Soil Amendments on Bacterial Community Structures

Stage of cropping process, rather than soil amendments, has a greater impact on the variance in bacterial community structures (Fig. 3A). This effect was reported by Ramírez *et al*. [45] in a maize field under long-term conservation agriculture management. Different soil amendments also had significant effects on soil bacterial community structures, but the variance in structure was weakened in the late maize cropping stage. A study had demonstrated that beta-diversity varied more across land uses than across time [46]. Our study indicated that the microbial community structures of the same agroecosystems tended to be more similar over time without the long-term soil amendment.

### Effects of Soil Amendments on the Microbial Composition Changes

The phyla *Acidobacteria*, *Actinobacteria*, *Alphaproteobacteria*, *Bacteroidetes*, and *Betaproteobacteria* were the abundant taxa in all soil samples, and were often present in the soil environment or shift from composting bacterial communities [47]. In autumn of the late maize cropping stage, *Bacteroidetes* were enriched in the composite soil amendments group. *Bacteroidetes* were reported to promote the mineralization of high-molecular-weight organic matter in soil [48]. This indicated that more *Bacteroidetes* were recruited to mineralize the large amounts of organic matter from composite soil amendments.

Our study found that rare and abundant sub-communities had a preference for the bacterial community composition regardless of soil treatments. *Firmicutes* and *Deltaproteobacteria* were the unique phylum in rare and abundant sub-communities, respectively. Li *et al*. [13] also reported different assembly patterns of rare and abundant bacterial sub-communities in rice rhizosphere soil under short-term nitrogen deep placement.

### Bacterial Community Distribution under Application of Different Soil Amendments

Two of the top 10 genera in relative abundance, *Ohtaekwangia* and *Terrimonas* were found to distinguish the Complex group from the other two groups. *Ohtaekwangia* was reported to be the predominant genera, and was identified as the keystone taxa in agricultural soil [49, 50]. *Terrimonas* was known to be beneficial for nutrient uptake, and was also positively correlated with soil-quality parameters [51, 52]. Both were in the top 10 genera in relative abundance, but were not enriched in the Simple group. These findings imply that the application in the Composite group enhanced the specific distribution of these microorganisms.

In the top 10 rare sub-community genera in relative abundance, *Niastella*, *Pedobacter*, *Lacibacter*, and *Flavisolibacter* were enriched in the Composite group, all from the *Bacteroidetes* phylum. Studies reported an enrichment of members of the *Bacteroidetes* in the rhizosphere of the wild relatives as compared to their domesticated counterparts [53]. These findings suggested that *Bacteroidetes* of wild relatives may enrich in maize rhizosphere and promote plant growth through mineralization. Furthermore, many rare sub-community genera with low relative abundance were also enriched in the Composite group, which indicated that rare taxa may formed unique community structures under application of composite amendments pressure.

### Correlation between Bacterial Communities and Soil Physicochemical Properties

Plant growth and crop yield depend on plants acquiring adequate nutrients from the soil [54]. In our study, the soil amendments improved newly created farmland soil fertility and physicochemical properties (Table 1). Soil organic matter and nitrogen played a key role in forming and stabilizing soil structure and nutrient recycling [55]. Our study determined that all applications of soil amendments significantly improved the OM and TN of newly created farmlands in the whole maize cropping process. Composite amendments significantly increased phosphorous and AK in soil, which suggested that composite amendments proved to be a better fertilizer for improving soil nutrients and properties [5].

**Fig. 7:**
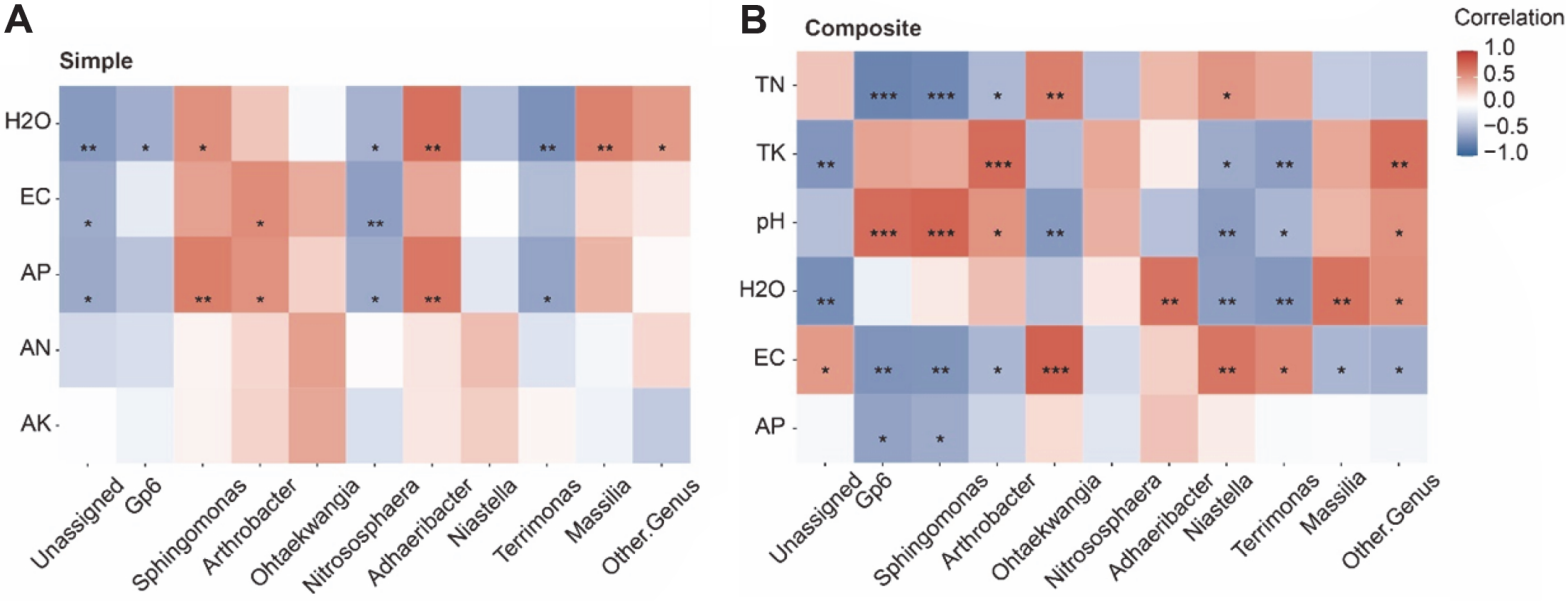
Correlation heatmap between bacterial genera and soil physicochemical properties. **A**. Correlation between bacterial genera and soil physicochemical properties under application of simple amendments. **B**. Correlation between bacterial genera and soil physicochemical properties under application of composite amendments.

Plant nutrition and soil quality were directly affected by the decomposition of organic matter, which in turn depends on soil microbiota [56]. Compared with the simple amendments group, more bacterial genera in the composite amendments group had stronger correlation with soil physicochemical properties (Fig. 7). These findings suggested that application of composite amendments may increase the diversity of soil microbiota and make them more active and functional when involved in organic matter decomposition.

*Ohtaekwangia* and *Niastella* had significant correlation with TN, EC, and pH under the application of composite amendments, but this was not observed in the Simple group. *Ohtaekwangia* and *Niastella* were reported to be the predominant taxa in the rhizosphere soil [49, 57], and *Ohtaekwangia* was related to the nitrogen cycling in soil [58]. These results indicated that *Ohtaekwangia* and *Niastella* may be enriched in maize root, and were involved in nitrogen and cycling of other matter after the application of composite amendments. Studies found that *Nitrososphaera* belong to Thaumarchaeota, which are known as ammonia oxidizers and prefer extreme environments [59]. However in our study, *Nitrososphaera* had significant negative correlation with moisture content, EC, and AP under the application of simple amendments. A correlation between *Nitrososphaera* and nitrogen or pH was not observed.

This study revealed the effects of different soil amendments on bacterial communities and soil physicochemical properties. In brief, the bacterial diversity, structure, and composition of rare and abundant sub-communities in soil had a specific distribution under the application of different soil amendments. The Chinese herb residues soil amendments with high nitrogen and organic matter additives significantly increased the relative abundance of rare taxa, which were more stable and increased the soil resiliency. Bacterial community structure was significantly affected by cropping systems and different soil amendments. Rare and abundant sub-communities demonstrated a preference for the bacterial community composition, with higher relative abundance of *Alphaproteobacteria* and *Betaproteobacteria* in abundant sub-community, and more *Bacteroidetes* in rare sub-community. Under the soil amendment with high nitrogen and organic matter additives, rare sub-communitiy genera *Niastella* and *Ohtaekwangia*, which were correlated with soil TN, EC, and pH, were enriched during the maize cropping process. *Nitrososphaera* was enriched under the application of simple amendments group soil, which had significant negative correlation with soil moisture content, EC, and AP. Applying soil amendments based on CHR could become an effective strategy to increase soil microbial diversity, improve microbial redundancy, and enhance soil fertility in the dry-farm agricultural practices of the Loess Plateau. However, the microbial communities characterized in this study were complex and dynamic with respect to the exogenous organic input and plant response. Different organic materials indicated the urgent need for further studies on this issue to identify and develop soil amendment combinations that maximize the utilization of Chinese herb residue in different agricultural systems.

## Figures and Tables

**Table 1:** Soil physical and chemical properties for the different soil treatments.

Spring	Treatment					*P*-value

Parameter	CK	CHR	CHR_U	CHR_SMS	CHR_MR	

OM (g/kg)	4.13 ± 0.21**a**	13.04 ± 0.6**b**	12.07 ± 1.03**b**	21 ± 1.47**d**	17.93 ± 0.56**c**	5.52E-09
TN (g/kg)	0.54 ± 0.09**a**	0.71 ± 0.04**a**	1.32 ± 0.11b**c**	1.1 ± 0.11**b**	1.37 ± 0.13**c**	3.83E-06
AN (mg/kg)	25.43 ± 0.59**a**	39.6 ± 0.63**b**	64.8 ± 0.37**c**	76.47 ± 0.97**d**	74.48 ± 1.04**d**	4.57E-15
TP (g/kg)	0.73 ± 0.06**a**	0.91 ± 0.06**b**	1.16 ± 0.04**c**	1.64 ± 0.08**d**	1.5 ± 0.07**d**	2.35E-08
OP (mg/kg)	17.49 ± 0.98**a**	22.19 ± 1.12**b**	20.8 ± 0.15**b**	27.29 ± 1.22**c**	27.37 ± 0.63**c**	3.51E-07
TK (g/kg)	28.64 ± 3.57**ac**	25.86 ± 0.58**a**	27.78 ± 1.1**ab**	30.89 ± 0.97**bc**	32.67 ± 0.63**c**	0.00646
AK (mg/kg)	98.74 ± 1.94**a**	138.4 ± 1.12**b**	134.35 ± 4.74**b**	190.28 ± 0.99**d**	161.18 ± 0.33**c**	7.05E-12
pH	8.21 ± 0.06**b**	7.94 ± 0.1**a**	8.02 ± 0.06**ab**	7.91 ± 0.12**a**	7.82 ± 0.1**a**	0.00318
EC (μs/cm)	10.03 ± 0.6**a**	9.91 ± 0.37**b**	10.65 ± 0.19**b**	10.59 ± 0.24**b**	10.75 ± 0.31**b**	7.19E-04
Moisture content (%)	0.26 ± 0.02**a**	0.41 ± 0.04**a**	0.39 ± 0.04**a**	0.4 ± 0.04**a**	0.37 ± 0.02**a**	0.0596

Autumn	Treatment					*P*-value

Parameter	CK	CHR	CHR_U	CHR_SMS	CHR_MR	

OM (g/kg)	5.56 ± 0.46**a**	12.1 ± 0.98**b**	11.45 ± 0.97**b**	21.67 ± 1.06**c**	19.84 ± 0.29**c**	1.85E-09
TN (g/kg)	0.65 ± 0.07**a**	0.96 ± 0.08**b**	1.04 ± 0.07**b**	1.46 ± 0.08**c**	1.48 ± 0.07**c**	3.12E-07
AN (mg/kg)	23.42 ± 0.43**a**	41.35 ± 0.99**b**	41.16 ± 1.02**b**	61.33 ± 1.03**c**	64.01 ± 4.84**c**	5.67E-09
TP (g/kg)	0.72 ± 0.05**a**	1.11 ± 0.14**b**	1.13 ± 0.11**b**	1.69 ± 0.06**c**	1.73 ± 0.05**c**	2.91E-07
OP (mg/kg)	11.88 ± 0.97**a**	19.58 ± 0.62**b**	17.82 ± 1**b**	29.58 ± 0.62**d**	25.67 ± 1.06**c**	2.34E-09
TK (g/kg)	30 ± 1.51**b**	27.54 ± 1.41**ab**	25.55 ± 1.02**a**	28.17 ± 0.13**ab**	27.5 ± 1.03**ab**	0.0101
AK (mg/kg)	134.86 ± 1.69**b**	144.7 ± 1.04**c**	122.71 ± 1.16**a**	186.22 ± 0.95**e**	165.56 ± 0.5**d**	7.87E-14
pH	8.17 ± 0.07**c**	7.87 ± 0.14**bc**	7.65 ± 0.12**ab**	7.43 ± 0.09**a**	7.35 ± 0.21**a**	1.07E-04
EC (μs/cm)	8.19 ± 0.47**a**	9.44 ± 0.51**a**	9.4 ± 0.45**a**	9.79 ± 0.37**b**	9.66 ± 0.25**b**	1.39E-06
Moisture content (%)	0.33 ± 0.04**a**	0.34 ± 0.02**b**	0.31 ± 0.02**b**	0.52 ± 0.02**b**	0.51 ± 0.03**b**	5.95E-03

All values were an average from three replicates ± SDs. The *p*-value of each row represented the statistical differences based on a one-way ANOVA test. Values with different letters within a row were significantly different based on Tukey’s multiple comparison test.

**Table 2:** The relative abundance of rare taxa for the different soil treatments.

Rare taxa relative abundance	Mean Diff.	95.00% CI of diff.	Adjusted *P*-value
CK_S vs. CHR_S	-20.75	-56.22 to 14.71	0.5879
CK_S vs. CHR_A	-15.77	-51.23 to 19.70	0.8646
CK_S vs. CHR_U_S	-32.28	-67.75 to 3.185	0.0957
CK_S vs. CHR_U_A	-14.28	-49.74 to 21.19	0.9195
CK_S vs. CHR_SMS_S	-36.92	-72.39 to -1.456	**0.0367**
CK_S vs. CHR_SMS_A	-38.40	-73.86 to -2.932	**0.0266**
CK_S vs. CHR_GM_S	-31.90	-67.36 to 3.568	0.1031
CK_S vs. CHR_GM_A	-41.97	-77.44 to -6.510	**0.0119**

All values were an average from three replicates ± SDs. The adjusted *p*-value of each row represented the statistical differences based on a Holm-Sidak multiple comparisons test.
